# ShrewdAttack: Low Cost High Accuracy Model Extraction

**DOI:** 10.3390/e25020282

**Published:** 2023-02-02

**Authors:** Yang Liu, Ji Luo, Yi Yang, Xuan Wang, Mehdi Gheisari, Feng Luo

**Affiliations:** 1 School of Computer Science and Technology, Harbin Institute of Technology (Shenzhen), Shenzhen 518055, China; 2 Peng Cheng Laboratory, Shenzhen 518066, China; 3 Guangdong Provincial Key Laboratory of Novel Security Intelligence Technologies, Shenzhen 518055, China; 4 Saveetha School of Engineering, Saveetha Institute of Medical and Technical Sciences, Chennai 602105, India

**Keywords:** model extraction attack, machine learning, MLaaS

## Abstract

Machine learning as a service (MLaaS) plays an essential role in the current ecosystem. Enterprises do not need to train models by themselves separately. Instead, they can use well-trained models provided by MLaaS to support business activities. However, such an ecosystem could be threatened by model extraction attacks—an attacker steals the functionality of a trained model provided by MLaaS and builds a substitute model locally. In this paper, we proposed a model extraction method with low query costs and high accuracy. In particular, we use pre-trained models and task-relevant data to decrease the size of query data. We use instance selection to reduce query samples. In addition, we divided query data into two categories, namely low-confidence data and high-confidence data, to reduce the budget and improve accuracy. We then conducted attacks on two models provided by Microsoft Azure as our experiments. The results show that our scheme achieves high accuracy at low cost, with the substitution models achieving 96.10% and 95.24% substitution while querying only 7.32% and 5.30% of their training data on the two models, respectively. This new attack approach creates additional security challenges for models deployed on cloud platforms. It raises the need for novel mitigation strategies to secure the models. In future work, generative adversarial networks and model inversion attacks can be used to generate more diverse data to be applied to the attacks.

## 1. Introduction

Machine Learning as a Service [1] helps customers benefit from machine learning by allowing developers to build quickly and efficiently using Machine Learning as a Service offerings, with access to pre-built algorithms and models without the cost, time, and risk associated with building an in-house machine learning team. According to a market report by Mordor Intelligence (https://www.mordorintelligence.com/industry-reports/global-machine-learning-as-a-service-mlaas-market (accessed on 27 December 2022)), the machine learning as a service market was worth $1.6 billion in 2020. It is expected to reach $12.1 billion by 2026, growing at a compound growth rate of 39.86% over the period 2021–2026. This is a very high growth rate, yet machine learning as a service platforms are at serious risk of data and model leakage.

Previous work [2,3,4] has shown that an attacker can access models on a platform solely through a black box and perform model extraction attacks using information such as model outputs, where the attack aims to gain task-specific capabilities of the targeted model under attack. We investigated a prevalent machine learning as a service provider, Microsoft Azure, which leverages two products, Azure Applied AI Services and Azure Cognitive Services, to provide trained models, which are available to application developers. The models are provided to application developers and end users on a pay-per-query basis. However, model extraction attacks can steal these well-trained models that can bring revenue to the platform and cause losses to the platform [5,6], which is the threat of model extraction attacks.

We focus on model extraction attacks in the black-box case, which is a more practical setup where the attacker has a general understanding of the task and data used by the target model, but no knowledge of the architecture of the target model, how it is trained, and the specific training data. The attacker can only obtain the model output by querying a large number of samples from the target model to build a substitute model training dataset to train the substitute model. Theoretically, more queries lead to better extraction attacks. However, a large number of queries will not only increase the query cost paid by the attacker to the platform, which is more expensive, but also easy to be restricted by the platform. Some studies show that the data distribution of some attacks that reach a certain number of queries is not consistent with the data distribution of normal queries. How to select more representative samples to query the target model and reduce the number of queries while being able to guarantee the availability of substitute models is a current research hotspot.

Model extraction attacks are also the process of building and training substitute models locally to obtain the functionality of the target model [7,8]. We want the substitute model to perform well on the task it is oriented to, with better usability and to be able to perform comparably to the target model, so we work on improving the performance of the substitute model for a specific task. This can work in two ways, on the one hand, choosing better architectures and training data for the local substitute models, and on the other hand, finding more effective ways of training the models to improve their performance.

We have studied these problems faced by model extraction attacks and proposed several ideas to reduce the cost of attacks and improve model performance in a more practical setting. Finally, we have proposed a complete model extraction framework. We make the following contributions:

- A general preprocessing step for model extraction attacks is proposed. We introduce the idea of instance selection into the attack process, which is particularly useful when an attacker can find a lot of data but wants to query only a few, and can make the most of this large data set while greatly reducing the amount of data to be queried.
- We propose the pre-trained model and the target model task-relevant data as a better choice for the substitute model architecture with training data, which reduce the queries and improve the performance of the substitute model. We also experimentally find it helpful to use semi-supervised learning of consistent regularization to train substitute models more efficiently, which can be learned from unlabeled data without querying the target model.
- We found the crucial role of model classification confidence in the attack. In the attack, we reduced the query by having the substitute model labeled before querying the target model, and we reduced the query by querying only the low-confidence data of the substitute model; after getting the output of the target model, we improved the efficiency and performance by training the substitute model using only the high confidence data of the target model.

We attacked two image classification models on Azure, a machine learning as a service provider, and achieved good experimental results with low cost and high accuracy, demonstrating the excellence of our solution.

## 2. Related Work

**Attack.** The concept of model extraction was first introduced by Tramer et al. [9]. They proposed a generic equation-solving attack for logical output layer models and a new decision tree path search algorithm. Ref. [10] showed that even querying the target model using random unlabeled problem domain or non-problem domain data gives good substitute results. It is shown in the paper again that such an approach has a high number of queries and costs a lot of time and money. Ref. [2] proposed a strategy for adaptive data sampling using hierarchies that improve the efficiency of querying the target model for the sampled data required. Ref. [11] focused on generating adversarial samples of the target model using substitute models. They generate synthetic datasets to query the target model, inspired by fast gradient symbolic attacks. This ensures that the substitute model is close to the decision boundary of the target model and requires very few queries. The substitute models are later used to generate adversarial samples that deceive the target model with a high probability. Ref. [3] utilizes active learning and unlabeled public datasets to perform model extraction, which improves query efficiency. However, the paper uses the same thief dataset for all tasks, querying a lot of unnecessary data, which is different from our approach, where we only query data relevant to the task. Ref. [12] explained the inherent limitations that prevent any learning-based strategy from extracting truly high-fidelity models, and in response to these limitations, they developed the first practical, functional equivalent model extraction that achieves high-fidelity extraction.

**Defense.** Ref. [13] proposed a cloud-based extraction monitor that quantifies the extraction status of a model by observing the query and response streams of individual and complicit adversarial users. Ref. [14] proposed PRADA, which can detect model extraction attacks against deep neural networks in a generic and efficient way. It analyzes the distribution of continuous API queries and alerts when this distribution deviates from benign behavior.

Ref. [15] proposed the first model watermarking framework for protecting image processing models. A uniform and invisible watermark is hidden in its output so that the hidden watermark will be learned and extracted when an attacker trains a proxy model using the input–output pairs of the target model. The robustness of the proposed watermarking mechanism is experimentally demonstrated, and it can resist substitute models trained with different network structures and loss functions. Ref. [16] proposed the Entangled Watermark Embedding (EWE), which forces the model to entangle legitimate task data and watermark representations, and showed that EWE is indeed robust to model extraction attacks by evaluating it on tasks from the visual and audio domains.

Ref. [17] utilizes a floating-point timing side channel to fully recover all parameters of the DNN model executed in software. Ref. [18] performs model extraction by learning the architecture-level execution characteristics of the kernel and the inter-layer temporal correlation information introduced by common DNN design practices. Ref. [19] makes the PCIe bus between the host and GPU devices a new attack surface to completely steal the entire DNN model, including the architecture, hyperparameters, and parameters. These are different from the models we attack: the models are provided by the Machine Learning as a Service platform and are only accessible in a black-box manner.

## 3. Threat Model

We consider a more realistic scenario where an attacker has the same ability to access models on the platform as a normal-use user. Unlike normal use, the attacker’s purpose is to perform a model extraction attack.

**Target models and their value.** The MLaaS platform provides a series of trained models for various tasks that are made available to users for use out-of-the-box. Users access these models through APIs, query the data, and obtain the output returned by the models. The platform earns revenue by charging users for their number of queries.

**Attacker’s capabilities.** The attacker has the same capabilities as a normal user. The user can:

- Know the task oriented by the target model, e.g., whether it is oriented to natural language processing or images, and if it is an image classification task, know whether it is a flower classification task or an animal classification task;
- Know the task for which the model is oriented, and the user can collect a dataset of waiting for query target models. For example, if the target model is a flower classification model, the user would find some flower images to query the target model and obtain the model’s output;
- Finally, the user uses this dataset to access the target model normally and obtain the model output for a particular purpose.

The user can only use the platform’s model normally after having these three points and obtaining enough information and data. Before an attacker is found by the platform to be attacking and is restricted, he or she should have these three capabilities as ordinary users. At the same time, the platform has restrictions that users are not allowed to know some contents that are often the core competencies of the platform’s models, including the following:

- The user does not know the architecture of the target model, linear model, or neural network and what architecture is used for the neural network;
- Not knowing the specific training data used by the target model;
- Not knowing the specific training method of the target model and model parameters.

**The attacker’s goal.** We focus on a more practical goal, where the attacker accesses the model in a normal way, obtains the model output, and builds a substitute model locally to obtain the decision capability. After obtaining the functionality of the target model, instead of paying to query the target model, it is sufficient to use this substitute model. The attacker can even provide this substitute model to others and get paid for it.

**Figure 1:**
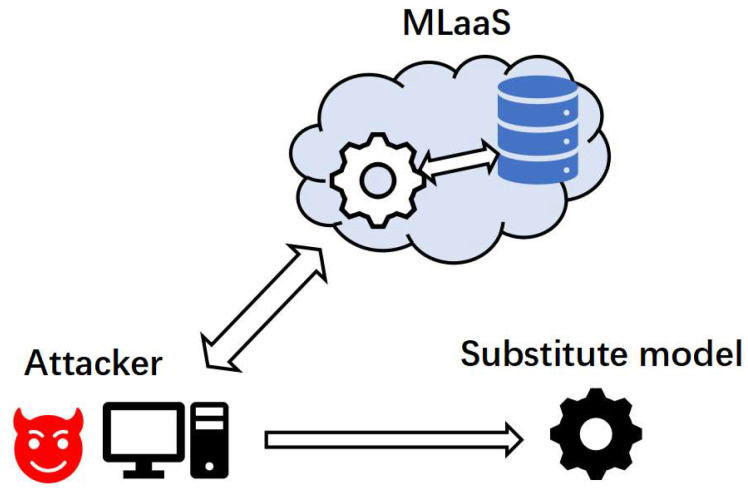
Overview of model extraction attack. MLaaS platforms train good models in the cloud using their data. The attacker uses its data to query the target model, obtains the returned output, and builds the substitute model locally.

Based on the threat model scenarios considered, the steps of our model extraction attack and the schematic diagram in Figure 1 are given as follows:

1. The attacker obtains the task oriented by the target model and chooses an architecture oriented to the same task for the substitute model;
2. The attacker knows what kind of data to query the target model with and finds data relevant to the task of the target model for the substitute model;
3. The attacker uses our proposed attack framework to carry out the attack based on the architecture and data identified for the substitute model. As mentioned earlier, our proposed framework can greatly reduce the data queried and spend less but can build a substitute model with high testing accuracy locally.

## 4. Our Framework

### 4.1. Using Data Relevant to the Target Model Task

We notice that some papers use data that are not relevant to the task of the target model, which can lead to many queries and cost more time and money, which is a waste. If the attacker does not know anything about the model, even if the target model is precisely replicated, he does not know how to use the obtained substitute model, so the attack is meaningless to the attacker. Instead of knowing nothing about the target model, the attacker can learn information about the target model in some way, such as the tasks that can be accomplished and the approximate type of data used. Only with some knowledge of the target model can one go for data relevant to the target model task. Using data relevant to the target model task eliminates the need to query irrelevant data, reducing the amount of data to be queried.

With some knowledge of the target model, the attacker can collect data on the web or use publicly available data; on the other hand, the attacker may also obtain some data that are used, such as in a real business. In our experiments, we use Google to search task-relevant data to approach the actual situation. The experiments achieve good results at a low cost.

### 4.2. Pre-Trained Models as Architecture

As mentioned above, the attacker can learn information about the target model, which the attacker can use to select a better architecture for the substitute model, i.e., a pre-trained model oriented to the target model task, in addition to using this information to find data [20]. We do not need to build a model from scratch, as doing so would require a lot of data, resources, and complex architecture. Instead, we could begin with a pre-trained model that has been trained on huge datasets and can predict various classes.

Pre-trained models are easily available. Many machine learning frameworks such as PyTorch, TensorFlow, and MXNet have a variety of pre-trained models for tasks such as image, text, and speech. For example, for image classification tasks, we can use a pre-trained image classification network that has learned to extract powerful and information-rich features from natural images as a starting point for learning new tasks. Most pre-trained networks are trained based on a subset of the ImageNet database used in the ImageNet Large-Scale Visual Recognition Challenge (ILSVRC). These networks have been trained on over one million images and can classify images into 1000 object classes, such as keyboards, coffee cups, pencils, and multiple animals. Generally, it is faster and easier to use pre-trained networks for migration learning than to train networks from scratch.

In a model extraction attack, the attacker can select a suitable pre-trained model as an architecture for a substitute model based on the task of the target model.

### 4.3. Data Reduction by Instance Selection

**Figure 2:**
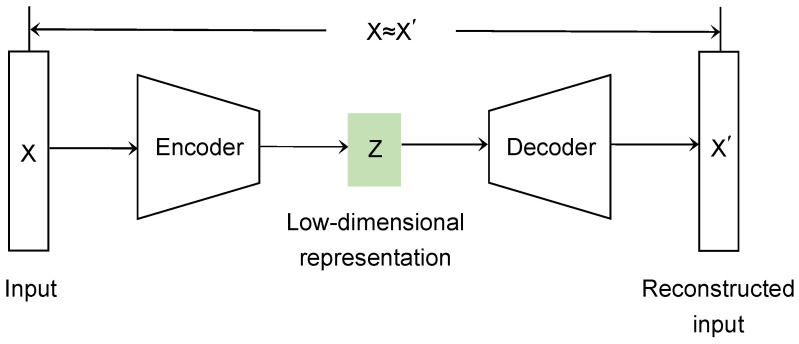
Autoencoder structure.

Instance selection aims to decide which instances in the training set should be retained for further use during the learning process [21,22]. By instance selection, the training set is reduced, which helps to reduce the running time during the training process. Applying the idea of instance selection to the model extraction attack effectively alleviates the problem of query overload. For our image classification task, the image data are first dimensionally reduced by an autoencoder [23,24,25], then clustered, and finally selected from the clustered data using a carefully designed algorithm to minimize duplicate or similar data and improve query efficiency. The structure of the autoencoder is shown in Figure 2.

We use the Mini-Batch K-Means algorithm [26] for clustering intermediate representations of encoders, a variant of the K-Means algorithm. The K-Means algorithm expects to optimize an objective function given the number of class centers of dataset *X*:

(1) $$\min\sum_{x\in X}{∥f\left(C,x\right)-x∥}^{2}$$

where C is the set of class centers and f(C,x) returns the class center c∈C that is closest to x. After selecting the initial center of mass, the algorithm does these two steps repeatedly: samples are assigned to their closest center of mass, and the new center of mass is computed using the average of all classes of samples. The algorithm stops when the difference between the old and new centers of mass is less than a threshold value.

The Mini-Batch K-Means algorithm has similar steps to the standard algorithm and still tries to optimize the same objective function. However, it randomly samples small batches in each training iteration, which greatly reduces the computation and computation time required for convergence and makes it faster for large data sets and potentially more robust to statistical noise. In practice, the difference in the quality of the results produced by this algorithm and the standard algorithm is usually tiny.

The data obtained after clustering are selected. First, the distance of each data to the class center of its class is calculated. For each class, there is a maximum distance. This maximum distance is divided into several distance segments, and then each piece of data belongs to a specific distance segment. Then, some data are picked randomly in each segment separately, and the data picked in each segment are proportional to the number of data falling in that segment. Finally, the data picked from all segments of all classes are aggregated for subsequent attacks. Such a picking method has three advantages. First, picking data within each distance segment reduces the use of similar or duplicate data, which ultimately reduces the query number of the attack; second, the data picked in each segment are proportional to the number of data falling in this segment, which will tend to pick more data from the segment with more data, ensuring data balance; third, after such a selection, when conducting attack experiments, we can prioritize the use of class center nearby data to train the substitute model, giving more importance to the data close to the class center. In addition, the data farthest from the class center are used last.

### 4.4. Substitute Models Query Only Low Confidence Data to Reduce Budget

Here, the attack aims to obtain the target model’s ability for a specific task. In our classification task, the model’s confidence in classifying a sample can be used to measure whether the model has gained the ability to classify this sample. We follow the approach of [27] and perform a softmax operation on the outputs of the substitute model if the outputs are not normalized. The normalized values are used in the metrics calculation to measure the model’s classification confidence. The softmax function is often used as the last activation function of a neural network to normalize the output of a network to a probability distribution. Probabilities are naturally more interpretable than real numbers in the range (−∞, +∞), which makes subsequent operations such as thresholding logical. Incorporating the idea into the attack process gives a method for selecting query samples.

**Figure 3:**
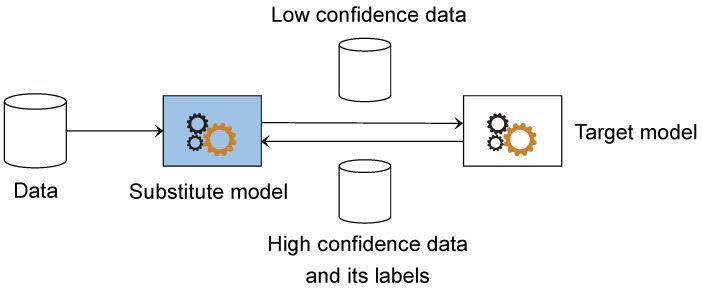
Query sample selection.

The data we have are first given to the substitute model for classification. The samples the model classifies with high confidence do not need to be labeled by the target model. It can be assumed that the model gains the ability to classify these samples, so only the samples that the model is not confident about are used to query the target model, where we reduce the number of queries. After the target model is labeled, the target model gives output that will serve as a classification target for the substitute model. However, not all labels returned by the target model are used. Only samples classified with high confidence by the target model and their results are used. The attack is to learn these abilities instead of the ability to classify even the target model with no confidence. Here, we further simplify the process and focus more on our task. The process of the method can be seen in Figure 3.

In this approach, a measure of model classification confidence is needed, so three metrics are proposed:

(2) $$P\left(y_{t,k_{1}}|x_{t}\right)\geq \tau$$

(3) $$P\left(y_{t,k_{1}}|x_{t}\right)-P\left(y_{t,k_{2}}|x_{t}\right)\geq \tau$$

(4) $$\sum_{i=1}^{n}P\left(y_{t,k_{i}}|x_{t}\right)\log P\left(y_{t,k_{i}}|x_{t}\right)\geq \tau$$

where $k_{i}$ is the i−th most confident class and τ is the threshold of confidence. Equation (4) is inspired by the concept of entropy, a measure of uncertainty. It can be seen that the larger the value of Equation (4), the greater the certainty, and it thus can be used as a measure of confidence in the model’s classification. Suitable metrics from these three should be selected according to the specific task and data characteristics.

### 4.5. Consistency Regularization

We propose a way to train the substitute model, which will train the two types of data in two different ways simultaneously, making the model’s training process more in line with the characteristics of the model extraction attack.

After the classification of the substitute model mentioned above, the low-confidence data are handed over to the target model for further labeling. After the results returned by the target model are obtained, this part of the data are still used to train the substitute model using cross-entropy loss supervision.

Note that, after classification by the substitute model, the high-confidence data are not labeled by the target model and cannot participate in the later supervised training. However, these data are still valuable for use. We hope the substitute model can continue to learn from this part of the data and improve its performance of the model, so we still train the substitute model with high-confidence data. We treat this part of the data as unlabeled data, and consistency regularization in semi-supervised learning is introduced and adapted [28,29]. Consistency regularization is designed to use the unlabeled data to train the model fully.

Specifically, for all the high confidence data of the substitute models involved in the training round, we calculate their losses in this way:

(5) $$w\left(t\right)\frac{1}{C|B|}\sum_{i\in B}{∥Z_{i}-{\tilde{Z}}_{i}∥}^{2}$$

(6) Z=αZ+(1−α)z

(7) $$\tilde{z}=Z/\left(1-\alpha^{t}\right)$$

where C is the number of classes, *B* is the set consisting of all high-confidence data involved in this training round, and *z* is the prediction vector given by this model. To subsequently combine the supervised and unsupervised loss terms, this part of the loss is scaled employing a time-dependent weighting function. At the same time, this approach aggregates multiple predictions previously evaluated by the network into an integrated prediction *Z*, where α is a momentum term that controls how close the ensemble output is to the training history, and *Z* thus contains a weighted average of predictions from the previous network. Such integrated predictions can be expected to predict unknown labels better than the network output at the most recent training round. They can therefore be used as a training targets. There can be less noise than using only one previous prediction. In order to generate training targets, we need to correct for startup bias in *Z* by dividing by a factor. Similar bias correction is used in Adam [30] and mean-only batch regularization [31]. In the first training round, the Z-sum was zero because there were no available data in the previous rounds.

To use consistent regularization, we need to note that two conditions should be satisfied. The first one is that the model needs to be trained with both labeled and unlabeled data, and this condition is satisfied. The second one is that the model being trained needs to have batch normalization or dropout. In our experiments, the substitute model is based on Squeezenet with these two components.

By using consistent regularization, we facilitate the full utilization of the data we can find. The later experimental section demonstrates that this improves the attack’s effectiveness and makes our extraction framework converge to perfection.

### 4.6. Complete Framework

**Figure 4:**
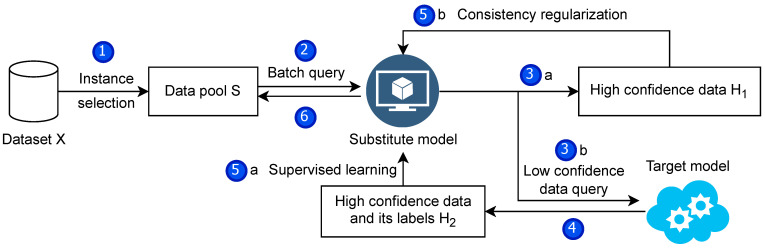
Attack framework. (1) The data we collected will be processed, and some samples will be selected using instance selection. (2) The training process will be divided into multiple substitution epochs. In each substitution epoch, a part of the data are extracted for the substitution model to label. In (3)a and (3)b steps, the high-confidence dataset and low-confidence dataset of the substitute models are obtained, respectively, and only the low-confidence dataset is queried for the target model. (4) leaves the high-confidence data of the target model and its labeled set. Finally, supervised learning in (5)a and consistent regularization in (5)b train the substitute models simultaneously.

We combine the previous methods and optimize the process to obtain a model extraction attack framework. As described in the relevant subsection, the framework fully considers the characteristics of the model extraction attack. It fully uses the training data while reducing the number of queries to the target model to reduce the query budget. The two parts of the training data used different training methods to train substitute models simultaneously in one go. Figure 4 illustrates the attack framework and its steps. In order to express the details of the framework algorithm in more detail, the algorithm’s pseudo-code is given in Algorithm 1.
**Algorithm 1 Model extraction attack algorithm.** Given a data set *X*, a substitute model $F_{\theta }$ with initial parameters θ, and a target model *G*, return a substitute model $F_{\theta^{\prime }}$ with similar functionality to *G*. Notice that the update of *Z* and $\tilde{z}$ can also be placed in the inner loop; here, it is placed outside for the sake of brevity:1:X←Instanceselection(X)2:$\theta^{\prime }\leftarrow \theta$3:**repeat**4:    D←Select(X,step)5:    step←step+Δ6:    $H_{1},L\leftarrow F_{\theta^{\prime }}\left(D\right)$▹ Query substitute model7:    $Z\leftarrow 0_{\left[\left|H_{1}\right|\times C\right]}$▹ Initialize the consistency regularization8:    $\tilde{z}\leftarrow 0_{\left[\left|H_{1}\right|\times C\right]}$9:    $H_{2}\leftarrow G\left(L\right)$▹ Leave high confidence data10:  **for** *t* in [*1*, *epochs*] **do**11:        $z_{i}\leftarrow F_{\theta^{\prime }}\left(x_{i}\in H_{1}\cup H_{2}\right)$▹ Train substitute model12:        $loss\leftarrow -\frac{1}{\left|H_{2}\right|}\sum_{x_{i}\in H_{2}}\log z_{i}\left[y_{i}\right]$▹ supervised loss13:        $\;\;+w\left(t\right)\frac{1}{C\left|H_{1}\right|}\sum_{x_{i}\in H_{1}}{∥z_{i}-{\tilde{z}}_{i}∥}^{2}$▹ unsupervised loss14:        update $\theta^{\prime }$ using, e.g., ADAM15:    **end for**16:    Z←αZ+(1−α)Z▹ accumulate ensemble predictions17:    $\tilde{Z}\leftarrow Z/\left(1-\alpha^{t}\right)$▹ bias correction18:**until** $F_{\theta^{\prime }}\approx G$19:**return** $F_{\theta^{\prime }}$

## 5. Evaluation

### 5.1. Experimental Setup

**Evaluation metric.** We define the purpose of the model extraction attack as obtaining the target model decision capability as much as possible, i.e., the substitute model should be as close as possible to or even exceed the performance of the target model on the test dataset, and this is carried out because we stand for a practical point of view and want the extracted model to have more satisfactory usability. Thus, we use such an evaluation metric—the ratio of the substitute model to the target model in test accuracy, as shown in the following equation, where $\hat{f}$ is the substitute model and *f* is the target model. This is different from the way some papers evaluate extraction attacks, Ref. [3] is more concerned with the proximity of the substitute model to the target model and how similar they behave on the data individuals; Ref. [11] is more concerned with the ability of the adversarial sample made using the substitute model to deceive the target model. They are more concerned with the indicator of the transferability of adversarial samples:

(8) $$\;\mathrm{Degree}\;\mathrm{of}\;\mathrm{substitution}\;=\;\mathrm{Accuracy}\;\left(\hat{f}\right)\;/\;\mathrm{Accuracy}\left(f\right)$$

**Target models and their data selection.** We use Microsoft’s Azure Custom Vision image recognition service, allowing users to build, deploy, and improve their own image identifier models. Image identifiers apply labels to images based on detected visual features, with each label representing a classification or object. We randomly selected 5000 image data from the training datasets of Intel-image and Fashion-MNIST, respectively, ensured a balanced number of data in each category, and trained two separate models using the service as targets for the attack. The models were trained on the platform for a few minutes. We needed to gain knowledge of the model architecture, weights, and hyperparameters. We could not change the models after training; only queries and the models would return output vectors. The trained model also gives the accuracy, which is 94.7% and 91% on the two datasets, respectively.

**Substitute models and their data selection.** The substitute model is based on Squee-zenet [32], a lightweight model that maximizes the speed of operations without losing network performance while reducing network parameters.

As we discussed in the threat model regarding the attacker’s capabilities, an attacker can collect a dataset of waiting queries for the target model oriented to that task based on the knowledge of the task the model is oriented to. We consider the following two types of data relevant to the target model’s task for the training dataset of the substitute model.

The first type of data comes from the validation set of Intel-image and part of the test dataset of Fashion-MNIST with 7301 data and 10,000 data, respectively. We strictly partitioned the datasets to ensure that the partial data from the validation set of Intel-image and the test dataset of Fashion-MNIST do not intersect with the training dataset of the two target models and the test dataset to be used later, respectively.

The second type of data comes from the image data relevant to the target model task we searched using Google. Some keywords relevant to the task are searched on Google. These include the specific type of data used by the target model and some other keywords relevant to the task. After the search, the images displayed by Google are obtained. Then, these data are downloaded using a bulk download tool. For an attacker, searching for specific data with Google is a better option than finding data from some large datasets because the desired data can be easily found, and the data quality is not low. We experimentally query the target model with a small portion of the Google search data to see how much data can be classified with high confidence by the target model. Using our metric for measuring model confidence, the percentage of high confidence classifications by the two target models for the two datasets obtained from the search is shown in Table 1. With the high percentage of high-confidence data in the table, the target models are very receptive to the data obtained from these searches. This indicates that the data searched with Google are usable, which is consistent with our direct observation that we found the data searched by Google to be of higher quality.

In our experiments, we considered two substitute model training datasets using a combination of the above two types of task-relevant data for each of the two target models.

The first substitute model training dataset consists of two types of task-relevant data. The ratio of the number of the two types is related to the percentage of the target model’s high confidence classification of the dataset obtained from the search in Table 1. This is handled this way because we are more concerned with the role of high-confidence data, and we want the substitute model to classify the high-confidence data of the target model with high confidence. Thus, we need to ensure that the number of data classified with high confidence by the target model is approximately equal in both relevant data types. At the same time, it can be assumed that the target model will classify a high percentage of the first data type with high confidence. The first substitute model training dataset is shown in Table 2.

The second substitute model training dataset consists of only the second type of data described above, i.e., using only task-relevant image data searched with Google. For Intel-image, the training dataset of the substitute model is 9149 data searched with Google. For Fashion-MNIST, the training data set of the substitute model is 30,626 data searched with Google. For Intel-image and Fashion-MNIST, the second substitute model training dataset is identified with $I_{2}$ and $F_{2}$, respectively.

The reason for considering the above two substitute model training datasets is that we want to explore the effect of data with different degrees of relevance to the target model task on the attack effectiveness. The effect of the attack is confirmed using the evaluation metrics described above. The first substitute model training dataset is more relevant to the target model task compared to the second substitute model training dataset.

To minimize queries and to be consistent with our proposed scheme, the substitute models are trained using five substitute epochs. Using the updated dataset, the substitute model is trained for 100 epochs in each epoch.

### 5.2. Experimental Results

**(1)**  **Effect of data with different degrees of correlation**

As we described, for Intel-image and Fashion-MNIST, the substitute models use two training datasets, respectively. $I_{1}$ is more relevant to the target model task than $I_{2}$, and $F_{1}$ is more relevant to the target model task than $F_{2}$. Table 3 shows this part of the experiment. We can find that the degree of data relevant to the task affects the performance of the substitute model, and the substitute model trained with data more relevant to the target model task performs better—not to mention that some papers use completely irrelevant data, which is a big waste because queries are paid for.

**(2)**  **The important role of high confidence data**

**Figure 5:**
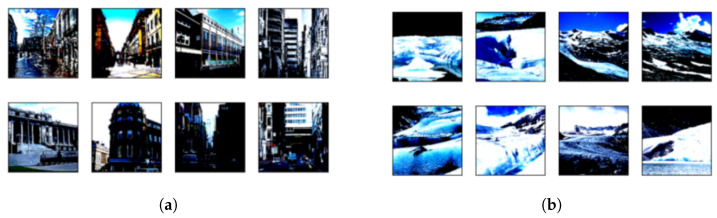
The substitute model confidently identifies two classes of data. (**a**) is data from the buildings class in the Intel-image dataset identified by the substitute model, and (**b**) is data from the glacier class in the Intel-image dataset identified by the substitute model. The untrained substitute model based on the pre-trained model identifies some of the data from these two classes with high confidence.

First, our method of evaluating model confidence is valid. In the experiments, the softmax function is applied to the output of the substitute model to calculate the probability, following the practice of existing work. When a component of the output after the action is greater than or equal to 0.9, the model is considered confidential in classifying that data. To demonstrate the validity of this measure, one can look at the training data of the untrained substitute model based on the pre-trained model SqueezeNet for classification confidence. Both figures in Figure 5 show the data filtered by the above measure of model confidence, clustered at a specific location of the components of the model output. These two classes of images correspond to exactly two classes of images. This demonstrates the usability of this method for measuring model confidence.

Second, the model trained with high confidence data only performs better on the test dataset than the model trained with all confidence data, as shown in Table 4.

**(3)**  **Effect of the ratio of labeled and unlabeled data**

**Figure 6:**
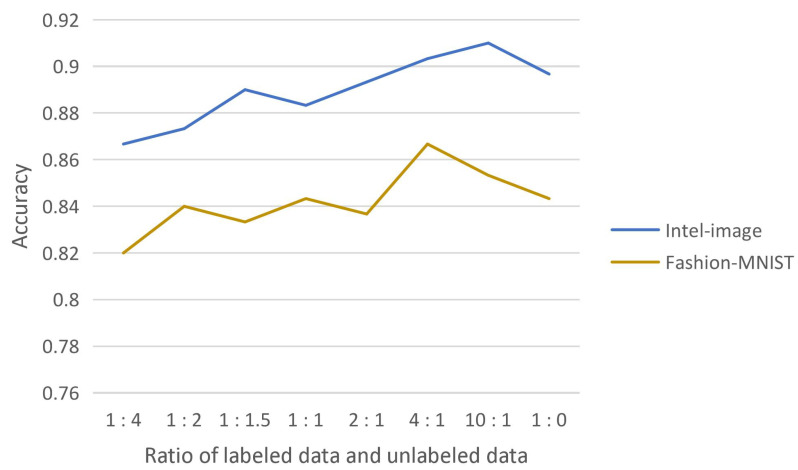
Effect of the ratio of labeled and unlabeled data on the accuracy of substitute model testing.

We experimentally demonstrate the effectiveness of introducing consistency regularization into the model extraction attack, which fully uses the substitute model’s high-confidence data while reducing the query to the target model. The substitute model effectively learns from this unlabeled data. The substitute model trained by adding this part of unlabeled data obtained the highest test accuracy compared to the substitute model trained with high-confidence data of the target model only. The effect of the ratio of labeled and unlabeled data on the test accuracy of the substitute model was also experimentally explored, as shown in Figure 6.

The reason for obtaining such results may be that the substitute model is trained with both labeled and unlabeled data and should learn knowledge mainly from supervised learning. More unlabeled data will interfere with the substitute model’s effective learning of labeled data, so more labeled data than unlabeled data are involved in the training. On the other hand, it is known from the experimental results that adding a small amount of unlabeled data are superior to completely using only labeled data, which indicates that the substitute model does learn knowledge from unlabeled data and the training of the substitute model needs the participation of unlabeled data.

**(4)**  **Extent of reduction of queries**

Our solution is dedicated to reducing queries and costs in several aspects. First, the found data are selected using the idea of instance selection. Two datasets, Intel-image and Fashion-MNIST, are reduced from 16,450 and 40,626 data to 3786 and 10,759 data, respectively. We also examined the case where, instead of using instance selection to pick, 3786 and 10,759 samples were randomly selected. The final impact of the two cases on the attack experiments is shown in Table 5. The test accuracies of the substitute models trained with the data selected using instance selection are 2.67% and 4.67% higher, respectively, compared to random selection, which proves the advantage of instance selection.

**Figure 7:**
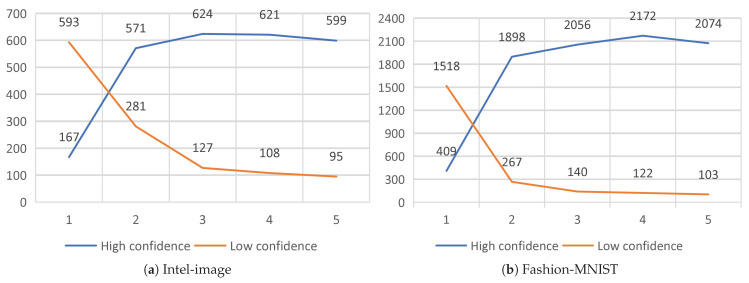
Degree of confidence in the data for the substitute models for each substitute epoch.

Second, the substitute model’s approach of querying only low-confidence data to reduce the budget greatly reduces the number of queries to the target model. To achieve the purpose of budget reduction, instead of querying all the training data of the substitute model at one time, we use five substitution epochs in batches to train the substitute model. Such an approach is beneficial to reduce the number of queries to the target model. As shown in Figure 7, the substitute model needs more confidence data in the first substitute epoch. After the training of the first substitute epoch, the classification ability of the substitute model is greatly improved. The confidence data are greatly increased, which means that only a small portion of the data needs to be used to query the target model, reducing the cost. At the same time, the data of the substitute model with confidence show an increasing trend, which illustrates the effectiveness of the training substitute model method.

Finally, we reduce the required queries from 16,450 and 40,626 to 1204 and 2150 on the Intel-image and Fashion-MNIST datasets, requiring only 7.32% and 5.30% of the original data to be queried, which significantly improves the efficiency and reduces the expense.

**(5)**  **Degree of substitution of substitute models**

Two target models trained on Azure using Intel-image and Fashion-MNIST, respectively, were attacked using our scheme. The degree of substitution of the obtained substitute models is shown in Table 6.

**(6)**  **Evade an advanced defense method**

A well-known defense method against model extraction attacks, PRADA, was proposed by Juuti et al. [14]. They observed that (1) benign queries to the target model by normal users tend to match or approach a normal distribution and (2) the query samples used by attackers are deliberately generated and selected in order to detect the most information, and thus the query sequences used tend to deviate from a normal distribution.

For a query sequence $X=\left\{x_{0},x_{1},\ldots \right\}$ and the corresponding query result $Y=\left\{y_{0},y_{1},\ldots \right\}$, PRADA first calculates the distribution of distances $d_{i}$:

(9) $$d_{i}=\min_{j<i,y_{j}=y_{i}}∥x_{i}-x_{j}∥$$

The Shapiro–Wilk Test (a normal distribution test) is then used to determine whether it is a potential attack sequence.

**Figure 8:**
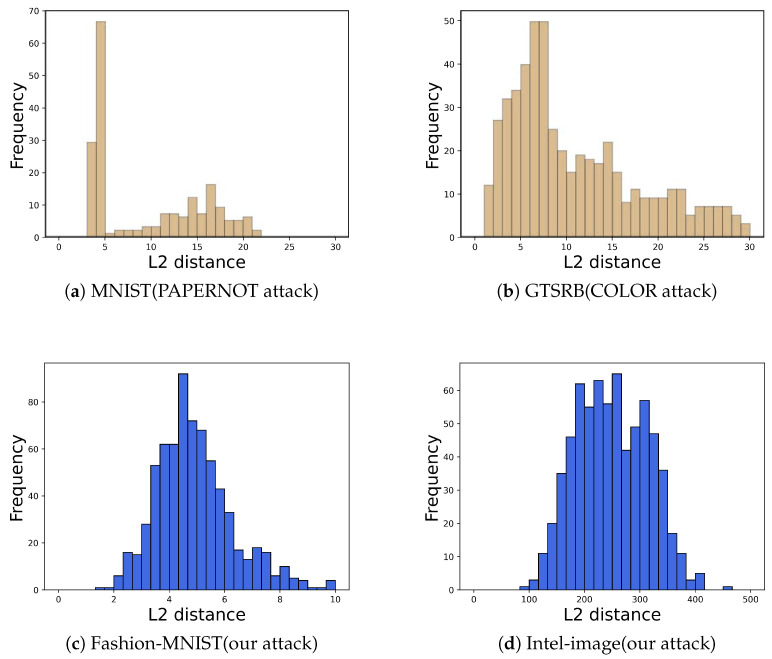
Distribution of query sequences. (**a**,**b**) are from [14]. (**a**) is the PAPERNOT attack query sequence for MNIST. (**b**) is the COLOR attack query sequence for GTSRB. (**c**) is our attack query sequence for FASHION-MNIST. (**d**) is our attack query sequence for Intel-image (the L2 distance is larger than the other experiments because of the larger size of the cropped images when preprocessing the Intel-image dataset). Our query sequence is closer to the normal distribution.

We applied PRADA to detect our attack and obtained histograms of the distribution of query sequences $d_{i}$ for the two datasets Intel-Image (PRADA hyperparameters δ = 0.91) and Fashion-Mnist (PRADA hyperparameters δ = 0.86) shown in Figure 8. The results show that our query sequence $d_{i}$ corresponds to an essentially normal distribution. Thus, PRADA does not detect our attack. This is because our attack uses natural image data to query the target model instead of using synthetic samples to query the target model.

## 6. Conclusions

This paper proposed a novel model extraction framework to reduce the cost of attacks and improve substitute models’ performance in a more practical setting. In particular, we use task-relevant data and pre-trained models of the target model as a better choice for substitute model training data and architecture. We use an instance selection method to select representative data in the dataset for the subsequent attack, reducing the use of data. In the attack, the substitute model queries only its low-confidence data, reducing queries; it also trains the substitute model using only the high-confidence data of the target model, significantly improving the model’s performance. For substitute models’ high-confidence data, which are unlabeled, we use consistency regularization to exploit these valuable data, which further enhances the attack. In addition, we use multiple substitution epochs for the attack. Such an approach fits well with the characteristics of model extraction attacks and can effectively reduce the query budget. We incorporate the above methods in a general model extraction framework and experimentally validate the framework’s effectiveness. The results show that the substitute models achieved 96.10% and 95.24% accuracy while querying only 7.32% and 5.30% of their training data on two target models, respectively. In future work, some techniques, such as generative adversarial networks and model inversion attacks, can be applied to the framework to generate more diverse query data to improve the attacks’ performance.

## Figures and Tables

**Table 1:** High confidence ratio of the target model on the data searched by Google.

Dataset	Total Amount of Data	Data of High Confidence Classification	Percentage of High Confidence Data
Intel-image	519	408	78.61%
Fashion-MNIST	1658	501	30.22%

**Table 2:** The first substitute model training dataset. For Intel-image and Fashion-MNIST, the first substitute model training dataset is identified with $I_{1}$ and $F_{1}$, respectively.

Dataset	Type I Data	Type II Data	Ratio of the Two Types of Data	Total Amount of Data
$I_{1}$	7301	9149	1:1.25	16,450
$F_{1}$	10,000	30,626	1:3.06	40,626

**Table 3:** Effect of data with different degrees of correlation.

Dataset	Substitute Model Training Dataset	Substitute Model Test Accuracy
Intel-image	$I_{1}$	91%
	$I_{2}$	88.33%
Fashion-MNIST	$F_{1}$	86.67%
	$F_{2}$	84%

**Table 4:** Impact of different confidence data.

Dataset	Substitute Model Training Dataset	High Confidence Data	All Confidence Data
Intel-image	$I_{1}$	91%	87%
	$I_{2}$	88.33%	83.33%
Fashion-MNIST	$F_{1}$	86.67%	82%
	$F_{2}$	84%	78.67%

**Table 5:** Accuracy of substitute models under instance selection and random selection.

Dataset	Instance Selection	Random Selection
Intel-image	91%	88.33%
Fashion-MNIST	86.67%	82%

**Table 6:** Degree of substitution.

Dataset	Substitute Model Training Dataset	Substitute Model Test Accuracy	Target Model Test Accuracy	Degree of Substitution
Intel-image	$I_{1}$	91%	94.7%	96.10%
	$I_{2}$	88.33%	94.7%	93.27%
Fashion-MNIST	$F_{1}$	86.67%	91%	95.24%
	$F_{2}$	84%	91%	92.31%

**Figure A1:**
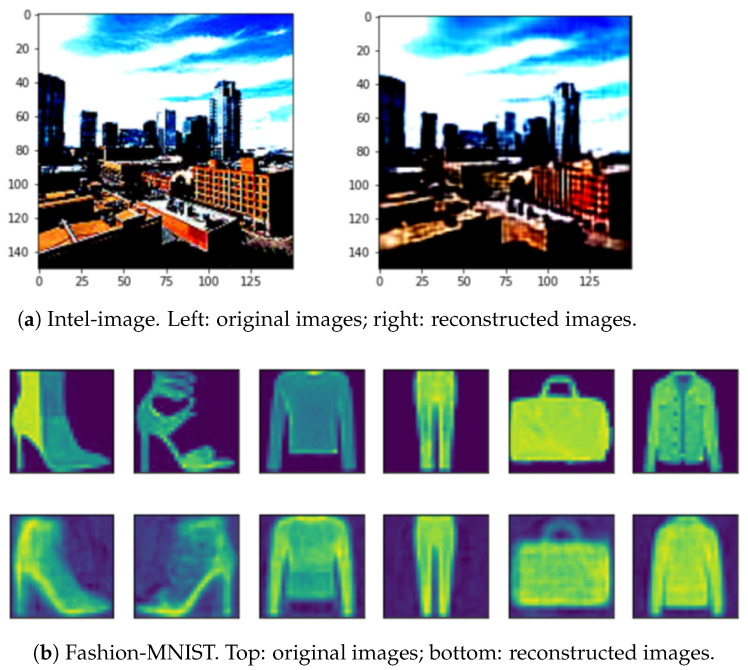
Reconstructions of the first autocoder.

## Data Availability

Publicly available datasets were analyzed in this study. These data can be found here: https://www.kaggle.com/datasets/puneet6060/intel-image-classification and https://www.kaggle.com/datasets/zalando-research/fashionmnist (accessed on 27 December 2022).

## Appendix A. Autoencoder

In reducing the query data by instance selection, we constructed two autoencoders to reduce the dimensionality of the image data using autoencoders. The first autoencoder mainly uses convolutional and transposed convolutional layers to adapt to the characteristics of the image data. The reconstruction results of the first autoencoder for Intel-image and Fashion-MNIST are shown in Figure A1. The second autoencoder uses fully connected layers further to compress the intermediate representation of the first one. For the Intel-image dataset, the structures of the two autoencoders are shown in Table A1, Table A2, Table A3 and Table A4.

**Table A1:** Encoder of the first autoencoder.

Layer (Type)	Convolution Kernel	Output Shape	Param #
Input		[3, 150, 150]	-
Conv2d-1	(11, 11)	[96, 36, 36]	34,944
ReLU-2		[96, 36, 36]	0
Conv2d-3	(5, 5)	[256, 34, 34]	614,656
ReLU-4		[256, 34, 34]	0
Conv2d-5	(3, 3)	[384, 32, 32]	885,120
ReLU-6		[384, 32, 32]	0
Conv2d-7	(3, 3)	[128, 32, 32]	442,496
ReLU-8		[128, 32, 32]	0
Conv2d-9	(3, 3)	[16, 30, 30]	18,448
ReLU-10		[16, 30, 30]	0
Conv2d-11	(3, 3)	[8, 30, 30]	1160
ReLU-12		[8, 30, 30]	0

**Table A2:** Decoder of the first autoencoder.

Layer (Type)	Convolution Kernel	Output Shape	Param #
Input		[8, 30, 30]	-
ConvTranspose2d-1	(4, 4)	[16, 31, 31]	2064
ReLU-2		[16, 31, 31]	0
ConvTranspose2d-3	(4, 4)	[128, 32, 32]	32,896
ReLU-4		[128, 32, 32]	0
ConvTranspose2d-5	(5, 5)	[384, 34, 34]	1,229,184
ReLU-6		[384, 34, 34]	0
ConvTranspose2d-7	(5, 5)	[256, 36, 36]	2,457,856
ReLU-8		[256, 36, 36]	0
ConvTranspose2d-9	(6, 6)	[96, 37, 37]	884,832
ReLU-10		[96, 37, 37]	0
ConvTranspose2d-11	(10, 10)	[3, 150, 150]	28,803

**Table A3:** Encoder of the second autoencoder.

Layer (Type)	Output Shape	Param #
Input	[7200]	-
Linear-1	[4096]	29,495,296
ReLU-2	[4096]	0
Linear-3	[1024]	4,195,328
ReLU-4	[1024]	0
Linear-5	[256]	262,400
ReLU-6	[256]	0
Linear-7	[16]	4112

**Table A4:** Decoder of the second autoencoder.

Layer (Type)	Output Shape	Param #
Input	[16]	-
Linear-1	[256]	4352
ReLU-2	[256]	0
Linear-3	[1024]	263,168
ReLU-4	[1024]	0
Linear-5	[4096]	4,198,400
ReLU-6	[4096]	0
Linear-7	[7200]	29,498,400
